# Association of Cholecystectomy with the Risk of Prostate Cancer in Patients with Gallstones

**DOI:** 10.3390/cancers12030544

**Published:** 2020-02-27

**Authors:** Chien-Hua Chen, Cheng-Li Lin, Chia-Hung Kao

**Affiliations:** 1 Digestive Disease Center, Changbing Show-Chwan Memorial Hospital, Lukang Township, Changhua County 500, Taiwan; showchench@yahoo.com.tw; 2 Digestive Disease Center, Show-Chwan Memorial Hospital, Changhua 500, Taiwan; 3 Department of Food Science and Technology, Hungkuang University, Taichung 433, Taiwan; 4 Management Office for Health Data, China Medical University Hospital, Taichung 404, Taiwan; orangechengli@gmail.com; 5 College of Medicine, China Medical University, Taichung 404, Taiwan; 6 Graduate Institute of Clinical Medical Science, School of Medicine, College of Medicine, China Medical University, No. 2, Yuh-Der Road, Taichung 404, Taiwan; 7 Department of Nuclear Medicine and PET Center, China Medical University Hospital, Taichung 404, Taiwan; 8 Department of Bioinformatics and Medical Engineering, Asia University, Taichung 404, Taiwan

**Keywords:** Cholecystectomy, Prostate cancer, Gallstones

## Abstract

*Objectives:* To assess the association of cholecystectomy with the risk of prostate cancer in patients with gallstones. *Methods:* This nationwide population-based cohort study was conducted by retrieving the Longitudinal Health Insurance Research Database (LHID2000) for inpatient claims in the Taiwan National Health Insurance (NHI) program. The study cohort consisted of 72,606 men aged ≥ 20 years with gallstones undergoing cholecystectomy between 2000 and 2010. The control cohort consisted of the men with gallstones, but without cholecystectomy, by 1:1 propensity score matching with the study cohort based on age, sex, urbanization, occupation, comorbidities, and the index date. We compared the hazard ratio of prostate cancer between both of the cohorts. *Results:* The incidence of prostate cancer was 0.76/1000 person-years for the non-cholecystectomy cohort and 1.28/1000 person-years for the cholecystectomy cohort [aHR (adjusted hazard ratio) = 1.67, 95% confidence interval (CI = 1.45–1.92), respectively (*p* < 0.001). When compared with the non-cholecystectomy cohort, the hazard ratio of prostate cancer for the cholecystectomy cohort was 1.49-fold greater (95% CI = 1.04–2.11) for follow-up ≤ 1 year, 1.52-fold greater (95% CI = 1.24–1.86) for follow-up 1–5 years, and 1.99-fold greater (95% CI = 1.56–2.53) for follow-up > 5 years, respectively. *Conclusions:* Cholecystectomy is associated with an increased hazard ratio of prostate cancer in gallstones patients, and the risk increases with an incremental period of follow-up. This observational study cannot ascertain the detrimental mechanisms of cholecystectomy for the development of prostate cancer, and cholecystectomy is not recommended for the prevention of prostate cancer based on our study.

## 1. Strengths and Limitations of This Study

1. The findings of this population-based cohort study provide the generalizability to Taiwan, since they retrieve the database from a 12-year-long follow-up of a mandatory National Health Insurance Program of Taiwan, with a coverage for 99.2% of Taiwan residents.
2. We compare the subsequent hazard ratio of prostate cancer between gallstones patients with and without cholecystectomy, rather than between patients with and without gallstones, to diminish the surveillance bias.
3. We also assess the incidence and hazard ratio of prostate cancer based on the follow-up period to assure that the findings of this study remain constant over time.
4. This observational study cannot provide causal pathogenesis between cholecystectomy and prostate cancer and several potential confounding factors are inherently unavailable in the database.

## 2. Introduction

Prostate cancer ranks as the top commonest malignancy for men in Western countries, with 28% and 22.2% of newly reported prostate cancers for American men in 2010 and for European men in 2008 [1,2]. Prostate cancer for men is less prevalent in Asia, but its incidence in Taiwan has increased from 7.5/100,000 to 23.5/100,000 between 1992 and 2007 [3]. In addition to aging and family history, metabolic syndrome has been supposed to enhance the development of prostate cancer [4,5]. However, the etiology of prostate cancer remains multifactorial and undetermined. The global incidence of prostate cancer increases steadily; therefore, it is important to identify the potential predictors of prostate cancer.

Gallstone disease is the most common disease for hospitalization in the gastrointestinal department, with reported gallstones prevalence of 10–15% in the United States and 5% in Taiwan for the adults [6,7,8,9]. The global prevalence of gallstones increases steadily due to sedentary lifestyle, westernization of dietary habits, and increased aging population [8]. Cardiovascular disease is the most commonly mentioned extra-biliary manifestation of gallstones in recent studies. The causal relationship between cardiovascular disease and gallstones might be bidirectional, but it remains undetermined, since the literature has shown the risk of cardiovascular disease might increase or decrease after cholecystectomy and atherosclerosis has been shown to be capable of impairing gallbladder emptying [10,11,12,13,14]. Former epidemiologic studies have shown that gallstones disease is related to the development of prostate cancer [15,16,17,18]. Shared risk factors or increased oxidative stress in both gallstones and prostate cancer may be the possible pathophysiological mechanisms for the aforementioned association [4,8,9,19,20]. In addition to cholesterol gallstones formation resulting from bile supersaturation, the presence of abundant cholesterol deposition in the prostate can enhance the development of prostate cancer [17,21]. However, whether gallstone disease is a risk factor or an epiphenomenon of prostate cancer remains debated. Moreover, most patients with gallstones remain asymptomatic and surveillance bias is raised about the former literature, as some patients with asymptomatic gallstones will be misclassified into non-gallstones subjects and the patients with known gallstones may obtain more accessibility for prostate cancer screening [22].

In this study, we hypothesized that the risk of prostate cancer will decrease after cholecystectomy if gallstone disease *per se* is a risk factor, rather an epiphenomenon, of prostate cancer. We utilized the Longitudinal Health Insurance Research Database (LHID2000) of the Taiwan National Health Insurance Research Database (NHIRD) to compare the hazard ratio of prostate cancer between gallstones patients with and without cholecystectomy, rather than between patients with and without gallstones, and the surveillance bias can be diminished.

## 3. Methods

### 3.1. Data Source

The mandatory NHI program has been launched since 1 March 1995 and it has covered approximately 99.2% of the 20 million residents in Taiwan. This retrospective nationwide population-based cohort study utilized the LHID2000 of the NHIRD database for inpatient claims in the National Health Insurance (NHI) program of Taiwan [23]. Our former studies have thoroughly detailed the NHI program and LHID2000 [24,25]. Furthermore, we used the 2001 International Classification of Diseases, Ninth revision, Clinical Modification (ICD-9-CM) for the claim codes.

### 3.2. Ethics Statement

To protect privacy, the patient personal information has been encrypted in the NHIRD. A non-profit organization, the National Health Research Institute (NHRI) of Taiwan, was in charge of the database of NHIRD, and the researchers could apply the relevant claims information for medical research with a waiver of patient consent. The Institutional Review Board (IRB) of China Medical University (CMUH-104-REC2-115-CR4) has approved this study.

### 3.3. Sampled Patients

**Figure 1:**
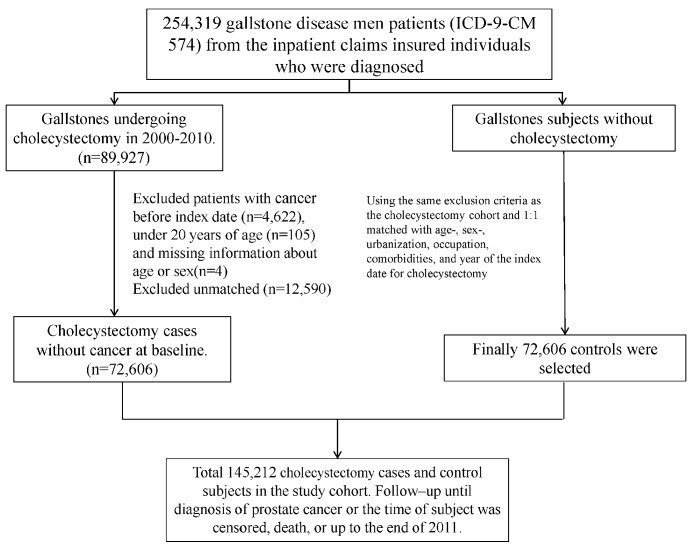
The selection process for the gallstones patients with cholecystectomy and without cholecystectomy.

We identified men aged ≥ 20 years with gallstones (ICD-9-CM 574) undergoing cholecystectomy between 2000 and 2010 from NHIRD as the control cohort. We randomly selected the control cohort with asymptomatic gallstones, and not undergoing cholecystectomy, from NHIRD by 1:1 propensity score matching with the case cohort based on age (every five-year span), sex, urbanization, occupation, comorbidities of hypertension, hyperlipidemia, chronic kidney disease, stroke, coronary artery disease, diabetes mellitus, chronic obstructive pulmonary disease, asthma, chronic kidney diseases, alcohol-related illness, colorectal polyps, congestive heart failure, benign prostatic hyperplasia, urolithiasis, urinary tract infection, and obesity, and the year of the index date for cholecystectomy. The same date of the matched patients was the reference index date appointed for the control patients. The patients with a history of malignancy (ICD-9-CM 140-208) or those with incomplete information of age or sex were excluded from the study. We followed up each patient from the index date until the development of prostate cancer (ICD-9-CM 185), death, withdrawal from the NHI program due to emigration, or the end of 2011. The deaths would be censored if the causes of deaths were unknown. Figure 1 shows the selection process for the gallstones patients with cholecystectomy and without cholecystectomy. We classified the urbanization levels based on the population density (people/km^2^), education level of residents, density of elderly and agricultural population, and the density of physicians [26]. Those with white-collar occupations comprised public servants, educators, or administrative personnel, who mainly engaged in indoor works. Those with blue-collar occupations comprised fishermen, farmers, or industrial laborers, who engaged in outdoor works with longer hours. Those with other occupations comprised the retired, jobless, or part-time working individuals.

### 3.4. Statistical Analysis

We analyzed the category variables by the chi-squared test and the continuous variables by the Student’s *t* test, respectively. We compared the cumulative incidence of prostate cancer by the Kaplan–Meier method and examined the differences by the log-rank test, respectively. We estimated the incidence of prostate cancer by stratifying age, occupation, urbanization, and presence or absence of comorbidity. We analyzed the adjusted hazard ratios (aHRs) and 95% confidence intervals (CIs) of prostate cancer in gallstones patients with and without cholecystectomy by the univariable and multivariable Cox proportional hazard regression models. Only those found to be significant in the univariable analysis were further examined in the multivariable analysis. The variables that were considered for multivariable analysis included age, gender, occupation, urbanization level, hypertension, hyperlipidemia, chronic kidney disease, stroke, coronary artery disease, diabetes mellitus, chronic obstructive pulmonary disease, chronic kidney diseases, alcohol-related illness, congestive heart failure, benign prostatic, hyperplasia, and test for prostate specific antigen. Moreover, we also assessed the incidence and hazard ratio of prostate cancer by dividing the duration of follow-up into ≤ 1 year, 1–5 years, and > 5 years, respectively. In Taiwan, the reported incidence of prostate cancer was about 0.30/1000 person-years in 2012; therefore, we measured the incidence of prostate cancer in 1000 person-years [27]. The proportional hazard model assumption was also examined while using a test of scaled Schoenfeld residuals. A SAS Version 9.4 (SAS Institute, Cary, NC, USA) was utilized for analysis and a two-tailed *p* < 0.05 was regarded as being statistically significant.

## 4. Results

The study identified a cholecystectomy cohort of 72,606 patients and a non-cholecystectomy cohort of 72,606 patients (Table 1). The mean ages in the cholecystectomy and non-cholecystectomy cohorts were 57.3 ± 15.8 and 57.4 ± 16.0 years, respectively. Most of the patients underwent cholecystectomy before aged 65 years (64.0%). The top three common comorbidities in the cholecystectomy cohort were hypertension (24.2%), diabetes (14.9%), and coronary artery disease (8.09%) in the order of the frequency. The number of patients receiving the test of prostate specific antigen tended to be greater in the cholecystectomy cohort (0.21% vs. p.11, *p* < 0.001). However, the presence of family history for prostate cancer was comparable between both of the cohorts.

**Figure 2:**
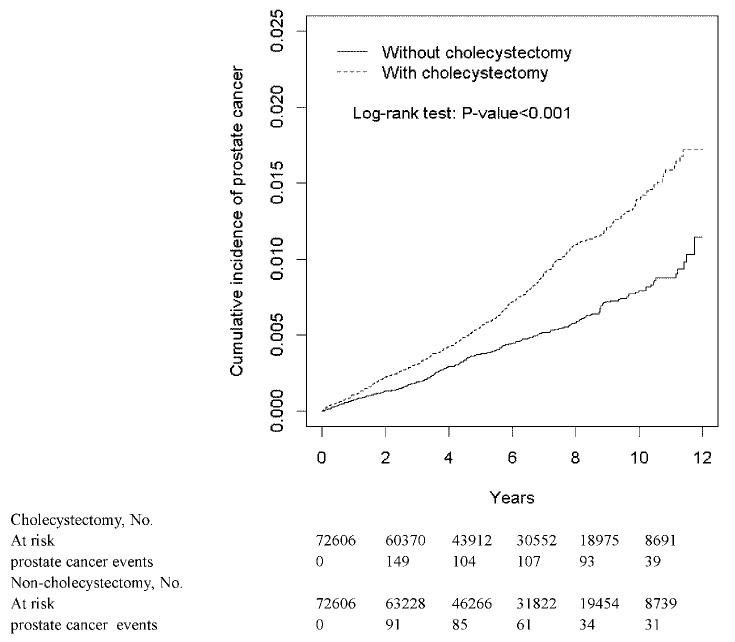
The cumulative incidence of prostate cancer in gallstones patients with cholecystectomy and without cholecystectomy.

Figure 2 shows that the cumulative incidences of prostate cancer were greater in the cholecystectomy cohort than those in the non-cholecystectomy cohort (log-rank test *p* < 0.001). The mean follow-up duration was 5.47 ± 3.25 years for the cholecystectomy cohort and 5.70 ± 3.13 years for the non-cholecystectomy, respectively (*p*-value < 0.001).

Table 2 compares the incidence of prostate cancer between gallstones patients with and without cholecystectomy based on the demographic characteristics and the presence or absence of comorbidity. The cholecystectomy cohort had a greater hazard ratio of prostate cancer when compared to the non-cholecystectomy cohort. The incidence rates of prostate cancer for the cholecystectomy and non-cholecystectomy cohorts were 1.28 and 0.76 per 1000 person-years, respectively (aHR = 1.67, 95% CI = 1.45–1.92). Except in the category of other occupations, the cholecystectomy cohort consistently had a greater incidence of prostate cancer in each stratification of age, occupation category, urbanization level, and the presence of comorbidity or not.

Table 3 compares the incidence of prostate cancer between the gallstones patients with and without cholecystectomy based on the stratification of follow-up period. The hazard ratio of prostate cancer increased with longer follow-up period for both cohorts. When compared with the non-cholecystectomy cohort, the hazard ratio of prostate cancer for the cholecystectomy cohort was 1.49-fold greater (95% CI = 1.04–2.11) for follow-up ≤ 1 year, 1.52-fold greater (95% CI = 1.24–1.86) for follow-up 1–5 years, and 1.99-fold greater (95% CI = 1.56–2.53) for follow-up > 5 years, respectively. In the model evaluating the hazard ratio for the development of prostate cancer throughout overall follow-up period, the results of the test revealed a nonsignificant relationship between Schoenfeld residuals for cholecystectomy and follow-up time, thus suggesting the proportionality assumption was not violated (*p*-value = 0.68).

## 5. Discussion

When compared with the reported incidence (0.30/1000 person-years) of prostate cancer for general population of Taiwan in 2012, the incidence of prostate cancer was greater for the gallstones patients in our study [27]. The main clinical complications of gallstones include biliary tract infection, pancreatitis, and biliary tract malignancy; and, cholecystectomy is mainly indicated for the presence of biliary complication, even though the threshold for cholecystectomy has declined in the era of laparoscopic cholecystectomy [22]. Our study shows that the removal of gallstones by cholecystectomy could not ameliorate the risk of prostate cancer, and the hazard ratio of prostate cancer in patients with gallstones would increase after cholecystectomy. Cholecystectomy is not recommended for prevention of prostate cancer based on the higher hazard ratio for prostate cancer after cholecystectomy in our study, which is in contrast with our hypothesis.

The hazard ratio of prostate cancer in gallstones patients increased with cholecystectomy in the multivariable Cox proportional hazard regression model. Moreover, except for the category of other occupations, the incidence of prostate cancer was consistently greater in the cholecystectomy cohort in each subgroup of age, occupation category, urbanization level, and the presence of comorbidity or not (Table 2). In addition, the deteriorated effect of cholecystectomy for the development of prostate cancer increased with longer follow-up even though the duration of follow-up was relatively short. The cholecystectomy cohort had shorter follow-up duration, but more cancer events were found in this cohort. (Figure 2 and Table 3). These findings confirm that cholecystectomy was associated with an increased risk of prostate cancer in patients with gallstones. However, we cannot ascertain the pathophysiological mechanism and the causal relationship in this observational study although cholecystectomy was generally indicated for biliary tract complication.

The exact pathophysiological mechanisms for the detrimental effect of cholecystectomy on prostate cancer remain unknown, but the possible explanations may be the following. First, cholecystectomy can enhance the risk of metabolic syndrome, which is also a modifiable risk factor for prostate cancer [4,8,9]. The removal of gallbladder will impair the enterohepatic circulation of bile acids; and, therefore, the gene expression that is related to the metabolism of bile acids, cholesterol, and glucose was hampered [28]. Second, cholecystectomy might increase oxidative stress and oxidative damage to DNA has gained attention to the development of prostate cancer [29]. Free radical reaction has been reportedly correlated to stone number and the severity of cholecystitis [30]. Moreover, the oxidative stress will deteriorate soon after abdominal surgery, although the long-term effect of cholecystectomy on the oxidative stress has never been mentioned [31]. Third, cholecystectomy might lead to gut dysbiosis, which is frequently observed in men with prostate cancer, even though its clinical implication for the pathogenesis is not well-known [32]. Through the alteration of the bile flow rhythm and the enterohepatic circulation of bile acids, the gut microbiota will be changed after cholecystectomy [33]. Moreover, our study is in agreement with the study from Shabanzadeh et al., to show that cholecystectomy cannot diminish the risk of prostate cancer [34]. Cholecystectomy has been shown to be capable of increasing the serum levels of cholecystokinin and secretin, and it is suggested to measure their serum levels before and after the removals of gallstones to ascertain whether this alteration is related to increased risk of prostate cancer [35,36].

There are several strengths in our study. First, our findings provide the generalizability to Taiwan, since we retrieve the database from a 12-year-long follow-up of a compulsory health care system with a coverage of 99.2% of Taiwan residents. Moreover, this is the first population-based cohort study on the association between cholecystectomy and prostate cancer. Second, we compare the subsequent hazard ratio of prostate cancer between gallstones patients with and without cholecystectomy, rather than between patients with and without gallstones to diminish the surveillance bias. Third, we also assess the incidence and hazard ratio of prostate cancer based on the follow-up period to assure our findings remain constant over time and diminish the bias of latent cancer as possible although the progression of prostate cancer is relatively insidious.

There are several limitations in our study. First, several potential confounding factors are inherently unavailable in the claims database, although the diagnosis of body mass index, smoking, and alcohol drinking has been replaced by obesity, chronic obstructive pulmonary disease, and alcohol-related illness, respectively. Second, we cannot individually review the accuracy of the claim data. However, the Taiwanese government should audit all of the insurance claims for medical reimbursement and a high concordance for the claims of diagnosis and utilization of medical resources has been validated in the literature [37,38]. The diagnosis of gallstones in Taiwan, either symptomatic or asymptomatic, should be made based on the presence of stones within the gallbladder through ultrasound, computed tomography, or magnetic resonance image. The asymptomatic gallstones patients without cholecystectomy were found during hospitalization for other medical illness, rather than biliary complications, and the presence of gallstones was coincidentally found by the image study. However, cholecystectomy is mainly indicated for symptomatic gallstones or gallstones-related biliary complications. The government audits the claim for cholecystectomy based on the indications and the acquisition of resected gallbladder specimens. Third, we mitigate the possible skewed association between cholecystectomy and prostate cancer due to surveillance bias by controlling for age, gender, occupation, urbanization level, comorbidities, test of elevated prostate specific antigen, and reimbursement claims for screening of prostate cancer due to family history. In Taiwan, there is no mass screening program, either by prostate specific antigen or ultrasound, for the asymptomatic men to detect prostate cancer [27]. Most physicians will only check prostate specific antigen for men with symptoms or family history of prostate cancer. Fourth, this observational study cannot provide causal pathogenesis between cholecystectomy and prostate cancer, and it requires more international studies to ascertain the environmental and ethnical generalizability.

## 6. Conclusions

Our findings demonstrate that cholecystectomy is associated with an increased risk of prostate cancer in patients with gallstones, and cholecystectomy might not be an appropriate option for those who have high risks of prostate cancer. Our study is subject to the inherent unavailability of some confounding factors in the claims database, and this observational study cannot ascertain the detrimental mechanisms of cholecystectomy for the development of prostate cancer.

## Figures and Tables

**Table 1:** Characteristics of patients between gallstone disease patients with cholecystectomy and without cholecystectomy.

Variables	Cholecystectomy				*p*-Value
	Yes		No		
	(*n* = 72,606)		(*n* = 72,606)		
	*n*	%	*n*	%	
Age, year					0.99
≤64	46,442	64.0	46,442	64.0	
65–74	14,936	20.6	14,935	20.6	
≥75	11,228	15.5	11,229	15.5	
Mean (SD) ^#^	57.3	15.8	57.4	16.0	0.20
Occupation					0.99
White collar	37,292	51.4	37,292	51.4	
Blue collar	25,611	35.3	25,611	35.3	
Others ^‡^	9703	13.4	9703	13.4	
Urbanization level ^$^					0.99
1 (highest)	20,354	28.0	20,351	28.0	
2	22,161	30.5	22,161	30.5	
3	12,270	16.9	12,271	16.9	
4(lowest)	17,821	24.5	17,823	24.6	
Comorbidity					
Hypertension	17,558	24.2	17,559	24.2	0.99
Hyperlipidemia	3035	4.18	3034	4.18	0.99
Stroke	4536	6.25	4539	6.25	0.99
Coronary artery disease	5874	8.09	5877	8.09	0.98
Diabetes mellitus	10,849	14.9	10,848	14.9	0.99
Chronic obstructive pulmonary disease	2798	3.85	2799	3.86	0.99
Asthma	1070	1.47	1069	1.47	0.99
Chronic kidney diseases	2178	3.00	2179	3.00	0.99
Alcohol-related illness	1386	1.91	1384	1.91	0.99
Colorectal polyps	367	0.51	367	0.51	0.99
Congestive heart failure	1022	1.41	1022	1.41	0.99
Benign prostatic hyperplasia	1337	1.84	1337	1.84	0.99
Urolithiasis	4104	5.65	4104	5.65	0.99
Urinary tract infection	2733	3.76	2733	3.76	0.99
Obesity ^†^	32	0.04	32	0.04	0.10
Test for prostate specific antigen	153	0.21	79	0.11	<0.001
Reimbursement claim ^†^	0	0.00	2	0.00	0.50

Chi-square test; ^#^: *T*-test; ^†^: Fisher-exact test. ^$^: The urbanization level was categorized by the population density of the residential area into 4 levels, with level 1 as the most urbanized and level 4 as the least urbanized. ^‡^: Other occupations included primarily retired, jobless, or part-time working individuals.

**Table 2:** Incidence and hazard ratio of prostate cancer between gallstone disease patients with cholecystectomy and without cholecystectomy.

Variables	Cholecystectomy						Crude HR (95% CI)	Adjusted HR (95% CI)
	Yes			No				
	Event	PY	Rate ^#^	Event	PY	Rate ^#^		
All ^1^	508	397,495	1.28	313	431,782	0.76	1.69(1.47, 1.95) ***	1.67(1.45, 1.92) ***
Age, year ^2^								
≤64	100	265,509	0.38	62	275,224	0.23	1.67(1.22, 2.30) **	1.66(1.21, 2.29) **
65–74	233	82,966	2.81	131	86,062	1.52	1.84(1.49, 2.28) ***	1.76(1.42, 2.18) ***
≥ 75	175	49,020	3.57	120	52,496	2.29	1.56(1.24, 1.97) ***	1.52(1.20, 1.91) ***
Occupation ^3^								
White collar	250	206,303	1.21	118	216,918	0.54	2.23(1.79, 2.78) ***	2.14(1.72, 2.67) ***
Blue collar	175	138,191	1.27	93	142,652	0.65	1.94(1.51, 2.49) ***	1.97(1.53, 2.54) ***
Others ^‡^	83	53,001	1.57	102	54,212	1.88	0.83(0.62, 1.11)	0.84(0.63, 1.12)
Urbanization level ^†^,^4^								
1 (highest)	157	112,576	1.39	90	118,910	0.76	1.84(1.42, 2.39) ***	1.73(1.33, 2.24) ***
2	144	121,228	1.19	109	126,281	0.86	1.38(1.07, 1.76) ***	1.36(1.06, 1.74) *
3	64	67,591	0.95	44	69,462	0.63	1.49(1.02, 2.19) *	1.54(1.05, 2.26) *
4(lowest)	143	96,100	1.49	70	99,129	0.71	2.10(1.58, 2.80) ***	2.16(1.62, 2.88) ***
Comorbidity ^&,5^								
No	205	239,386	0.86	110	247,935	0.44	1.93(1.53, 2.43) ***	1.96(1.56, 2.48) ***
Yes	303	158,109	1.92	203	165,846	1.22	1.57(1.31, 1.87) ***	1.51(1.27, 1.81) ***

PY, person-years; Rate ^#^, incidence rate per 1000 person-years; Crude HR, relative hazard ratio; Only those found to be significant in the univariable analysis were further examined in the multivariable analysis; ^1^ Adjusted HR was calculated by Cox proportional hazard regression and adjusted for age, gender, occupation, urbanization level, hypertension, hyperlipidemia, chronic kidney disease, stroke, coronary artery disease, diabetes mellitus, chronic obstructive pulmonary disease, chronic kidney diseases, alcohol-related illness, congestive heart failure, benign prostatic, hyperplasia, and test for prostate specific antigen; ^2^ Adjusted HR was calculated by Cox proportional hazard regression and adjusted for gender, occupation, urbanization level, hypertension, hyperlipidemia, chronic kidney disease, stroke, coronary artery disease, diabetes mellitus, chronic obstructive pulmonary disease, chronic kidney diseases, alcohol-related illness, congestive heart failure, benign prostatic, hyperplasia, and test for prostate specific antigen; ^3^ Adjusted HR was calculated by Cox proportional hazard regression and adjusted for age, urbanization level, hypertension, hyperlipidemia, chronic kidney disease, stroke, coronary artery disease, diabetes mellitus, chronic obstructive pulmonary disease, chronic kidney diseases, alcohol-related illness, congestive heart failure, benign prostatic, hyperplasia, and test for prostate specific antigen; ^4^ Adjusted HR was calculated by Cox proportional hazard regression and adjusted for age, occupation, hypertension, hyperlipidemia, chronic kidney disease, stroke, coronary artery disease, diabetes mellitus, chronic obstructive pulmonary disease, chronic kidney diseases, alcohol-related illness, congestive heart failure, benign prostatic, hyperplasia, and test for prostate specific antigen; ^5^ Adjusted HR was calculated by Cox proportional hazard regression and adjusted for age, gender, occupation, and urbanization level; ^†^: The urbanization level was categorized by the population density of the residential area into four levels, with level 1 as the most urbanized and level 4 as the least urbanized; ^‡^: Other occupations included primarily retired, unemployed, or low income populations; ^&^: Individuals with any comorbidity of hypertension, hyperlipidemia, chronic kidney disease, stroke, coronary artery disease, diabetes mellitus, chronic obstructive pulmonary disease, asthma, chronic kidney diseases, alcohol-related illness, colorectal polyps, congestive heart failure, benign prostatic hyperplasia, urolithiasis, urinary tract infection, and obesity were classified into the comorbidity group; * *p* < 0.05, ** *p* < 0.01, *** *p* < 0.001.

**Table 3:** Incidence and hazard ratio of prostate cancer between patients with cholecystectomy and without cholecystectomy stratified by follow-up period.

	Cholecystectomy						Compared to Patients without Cholecystectomy	
	Yes			No				
Variables	Event	PY	Rate ^#^	Event	PY	Rate ^#^	Crude HR (95% CI)	Adjusted HR ^§^ (95% CI)
Follow-up period								
≤1 year	75	70,486	1.06	53	71,829	0.74	1.44(1.01, 2.05) *	1.48(1.04, 2.11) *
1–5 years	231	208,578	1.11	159	219,186	0.73	1.53(1.25, 1.87) ***	1.52(1.24, 1.86) ***
>5 years	202	118,431	1.71	101	122,766	0.82	2.07(1.63, 2.63) ***	1.99(1.56, 2.53) ***

PY, person-years; Rate ^#^, incidence rate per 1000 person-years; Crude HR, relative hazard ratio; Only those found to be significant in the univariable analysis were further examined in the multivariable analysis; Adjusted HR ^§^ multivariable analysis including age, gender, occupation, urbanization level, hypertension, hyperlipidemia, chronic kidney disease, stroke, coronary artery disease, diabetes mellitus, chronic obstructive pulmonary disease, chronic kidney diseases, alcohol-related illness, congestive heart failure, benign prostatic, hyperplasia, and test for prostate specific antigen; * *p* < 0.05, *** *p* < 0.001.

## Data Sharing Statement

We access this dataset after approval of the Taiwan Ministry of Health and Welfare (MOHW). Email: stcarolwu@mohw.gov.tw; Address: No.488, Sec. 6, Zhongxiao E. Rd., Nangang Dist., Taipei City 115, Taiwan (R.O.C.); Phone: +886-2-8590-6848.

## Abbreviations

LHID2000	Longitudinal Health Insurance Research Database 2000
NHI	National Health Insurance
aHR	adjusted hazard ratio
CI	confidence interval
NHIRD	National Health Insurance Research Database
ICD-9-CM	International Classification of Diseases, Ninth Edition, Clinical Modification
NHRI	National Health Research Institute
IRB	Institutional Review Board
SDs	standard deviations
HRs	hazard ratios
